# Characterization of ductal carcinoma in situ cell lines established from breast tumor of a Singapore Chinese patient

**DOI:** 10.1186/s12935-014-0094-8

**Published:** 2014-09-20

**Authors:** Jacklyn WY Yong, Meng Ling Choong, SiFang Wang, Yu Wang, Shermaine QY Lim, May Ann Lee

**Affiliations:** Cell-Based Assay Development Group, Experimental Therapeutics Centre, Agency for Science Technology and Research, 31 Biopolis Way, #03-01 Nanos Building, Singapore, 138669 Singapore

**Keywords:** Immortalization, Telomerase, Breast cancer, Ductal carcinoma in situ, Tumorigenicity, Estrogen, Progesterone, Cell line, Chinese

## Abstract

**Background:**

Five cell lines were established from a Singaporean patient of Chinese origin with breast ductal carcinoma in situ (DCIS). These five cell lines express exogenous human telomerase reverse transcriptase (hTERT) which confers the ability to proliferate indefinitely.

**Methods:**

Cells were isolated from the DCIS excision and transfected with a plasmid expressing hTERT, a catalytic subunit of telomerase. Five immortalized colonies were propagated and characterized by karyotyping, array comparative genomic hybridization (CGH), immunostaining and Western blots for biomarkers, in vitro anchorage independent growth, in vivo mouse tumorigenicity, drug sensitivity, species authentication and virology safety testing.

**Results:**

Array CGH analysis showed that the cell lines harbored different specific genetic aberrations. Common mutations observed in most breast cancer cell lines such as the general loss of heterozygosity (LOH) throughout chromosome X and chromosome 17 are also observed in our cell lines. The cell lines were further characterized as human breast cells that are estrogen- and progesterone-receptor positive, and sensitive to tamoxifen. The cell lines showed anchorage-independent growth in the soft agar assay and can grow in common culture medium without supplementation with growth factor, therefore demonstrating transformed characteristics. Four of the cell lines can engraft and form measureable tumors after 50 days when injected subcutaneously into immune-deficient (SCID) mice. The weak tumorigenicity of these cell lines corresponded well with their non-malignant growth origin. The cell lines were authenticated to be of human origin based on DNA fingerprint of the cells. The cell lines were free from contamination of 20 viruses and mycoplasma in the virological safety test panel.

**Conclusions:**

Unlike most available breast cell lines, our cell lines are derived from primary breast cancer tissues that represent earlier grades or tumor progression stages. They would be useful as study models for basic and clinical research programs directed at early diagnosis and intervention.

## Background

There are many benefits in using primary cell cultures with little or no laboratory-induced transformation. The behaviors of these cells are hence more truly reflective of the organ of origins. However, the slow population doubling time and the finite lifespan of these primary cells hinder large scale experimentation that require continuous supply of cells with consistent behavior. Primary cultures require supplementation of growth factors and bovine pituitary extract which are costly and suffer from batch to batch variations. There is also ethical pressure on scientists to reduce the use of animal in laboratory research.

On the other hand, immortalized cell lines are the in vitro equivalent of cancerous cells as they maintained many characteristics of the cancer tissue from which it was derived. Cancer occurs when a somatic cell which normally cannot divide undergoes alterations which cause de-regulation of the normal cell cycle controls leading to uncontrolled proliferation. Immortalized cell lines have undergone similar alterations allowing a cell type which would normally not divide to proliferate in vitro*.* Cell lines continue to be used as models for medical research due to their ease of use and storage, and consistent cell behavior.

The most commonly used breast cancer cell line in the world is MCF-7. This, together with other commonly used breast cell lines such as the MDA-MB-series, are not derived from primary breast tumors but from tumor metastases in pleural effusions (reviewed in Burdall et al. [1]). Cells from metastatic tumors are often more aggressive than cells in the primary lesion. Results obtained through research work based on these cell lines should be interpreted as late-stage or higher grade breast cancers. However, current clinical practices lead to early diagnosis and treatment of breast cancers. Hence, there is a need for cell lines derived from primary tissues that represent the earlier grades of tumor progression stages. This would be more clinically relevant as most drug therapies are directed at these stages.

We have established five sub-lines derived from primary tissue obtained from a Singapore female patient with ductal carcinoma in situ, a common type of non-invasive breast cancer. The five sub-lines were obtained following over-expression of the human telomerase reverse transcriptase (hTERT) in primary cells. hTERT alone can immortalize cells without causing cancer-associated changes or altering phenotypic properties [2]. Establishment and characterization of these new cell lines were a lengthy process that took a couple of years to complete. Continuous cell lines have undergone significant mutations to become immortal. This can alter the biology of the cell and must be taken into consideration in any analysis. The recognized criteria of a bona fide continuous cell line [3] such as altered cyto-morphology, higher growth rate, reduced growth factor dependency, ability for anchorage independent growth, changes in ploidy, tumorigenicity and an infinite lifespan are documented in this report. These cell lines would be useful in drug screening of early stage breast cancer.

## Results

### Authentication and virology testing of ETCC001

**Figure 1:**
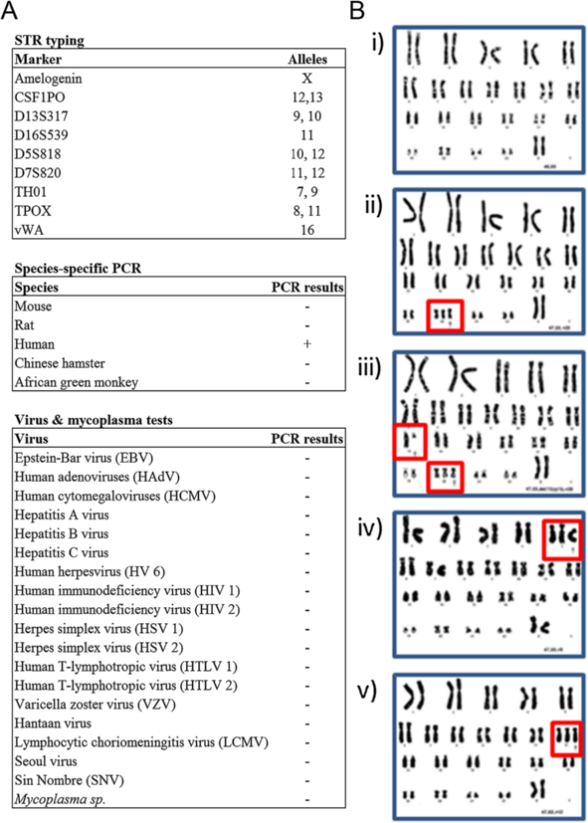
**Authentication and virological safety results for ETCC001. (A)** DNA fingerprinting using STR markers, species-specific PCR evaluation and virology study showed that ETCC001 is of human origin and free from 20 types of virus and mycoplasma contamination. **(B)** Karyotyping of ETCC001 breast cancer cells. Of 20 metaphases of ETCC001 analyzed, fourteen displayed normal female karyotype (i), four showed trisomy at chromosome 20 (ii and iii), and two exhibited non-clonal numerical abnormalities (trisomy at chromosome 5 and 12, iv and v, respectively). Giemsa/trypsin/Leishman (GTL) banding was used to obtain karyotype. +, presence of a PCR product; −, absence of a PCR product.

The Cancer Cell Isolation kit (Panomics) was used to isolate cancer cells from tissue obtained from a Singapore female with breast ductal carcinoma in situ. The resulting cell line was named ETCC001. ETCC001 cells were grown and maintained in M171 media. To verify the species origin of ETCC001, the cells were sent to IDEXX Laboratories, Minnesota, USA for STR DNA fingerprinting and PCR species evaluation. The alleles for nine different STR markers were established (Figure 1A). The result showed that ETCC001 is of human origin and is free from mammalian inter-species contamination (Figure 1B). ETCC001 is also not contaminated with the viruses tested (Figure 1C).

Karyotyping for ETCC001 was performed when the cells were at passage 40. Majority of the karyograms (14 out of 20 metaphases) depicted a normal karyotype (Figure 1D). Of the four karyograms showing trisomy 20, one demonstrated an aberration at chromosome 13 in addition to trisomy 20 (Figure 1D iii). In addition, metaphases containing trisomy 5 (Figure 1D iv) and 12 (Figure 1D v) were observed. The karyograms of ETCC001 confirmed that the cells were of female origin and that majority of the cells have normal karyotypes. However, clonal and non-clonal numerical chromosomal abnormalities were present, which probably provided the basis for the transformation and tumorigenesis.

### hTERT immortalization and cell transformation

**Figure 2:**
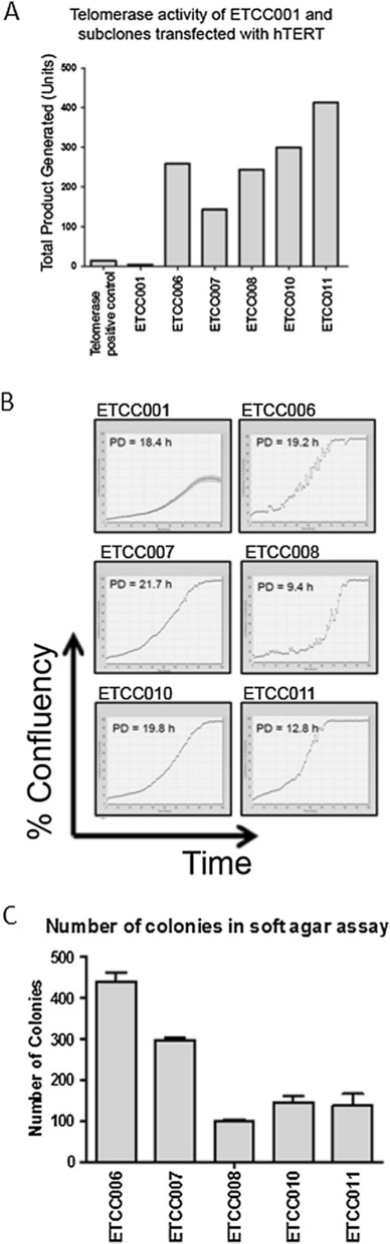
**Transfection of ETCC001 human breast cells with hTERT.** Five hTERT-transfected clones (ETCC006, ETCC007, ETCC008, ETCC010 and ETCC011) were obtained. **(A)** Telomerase activities were determined using TRAPeze-XL telomerase detection kit. Heat inactivated samples were used as negative controls and the readings were deducted as background. **(B)** Population doubling time (in hour) was determined using cell confluency as a measure of cell growth. Only log phase of the growth curve was used in the calculation of population doubling rate. **(C)** Colony counts of ETCC001 and hTERT-expressing cell lines in the soft agar assay. The assay was performed in triplicates and error bars represent standard deviation.

As M171 is a specialized medium used to culture primary human mammary cells, we determined whether ETCC001 could grow in RPMI supplemented with 10% FBS. However, ETCC001 was unable to grow in RPMI, suggesting that it was not transformed. Hence pGRN145 plasmid containing hTERT (telomerase reverse transcriptase, a catalytic subunit of telomerase) was transfected into ETCC001 cells. The pGRN145 plasmid contains a hygromycin resistance gene. Transfected ETCC001 cells were seeded in 15-cm plates. After two weeks, five hygromycin-resistant clones were obtained and named ETCC006, ETCC007, ETCC008, ETCC010 and ETCC011. There was a dramatic increase of telomerase activity in ETCC006, ETCC007, ETCC008, ETCC010 and ETCC011, compared to ETCC001 (Figure 2A). The clone ETCC011 showed the highest telomerase activity.

Over-expression of the TERT component of telomerase confers replicative immortality such that cells over-expressing it would continue to proliferate indefinitely [4]. To determine if the over-expression of hTERT and subsequent increase in telomerase activity can affect the population doubling rate and hence cell growth, the population doubling rates of the clones were determined. Figure 2B shows that ETCC001 can reach 50% confluency before growth stagnation while all the other cell lines can reach 100% confluency. A hallmark of transformed cells is the ability to grow to high density due to reduced contact-inhibition. In addition, the population doubling rates of the derived cell lines are similar to that of untransformed ETCC001, while ETCC008 and ETCC011 have distinct faster doubling rate compared to ETCC001. It should be noted that these transformed cell lines are capable of growth in RPMI1640 without growth factor supplements while ETCC001 can only grow in M171 medium supplemented with MEGS.

The average population doubling time of ETCC001 and the cell lines are about 20 hours (Figure 2B). The passage numbers of the cells were 131 for ETCC001, 93 for ETCC006, 86 for ETCC007, 53 for ETCC008, 83 for ETCC010 and 88 for ETCC011 at the time this manuscript was prepared. We passaged the cells every three to four days. This translates to more than 400 population doublings for ETCC001 and more than 200 population doublings for the five hTERT-expressing cell lines at the time of writing this manuscript.

Anchorage-independent growth is another hallmark of cell transformation and is the gold standard used to detect transformed malignant cells in vitro. All hTERT-transfected cells formed colonies in the soft agar with ETCC006 and ETCC007 forming the highest number of colonies (Figures 2C and D). ETCC001 did not form any colony in the soft agar assay, suggesting it has not acquired a transformed phenotype.

### Cell biomarkers

**Figure 3:**
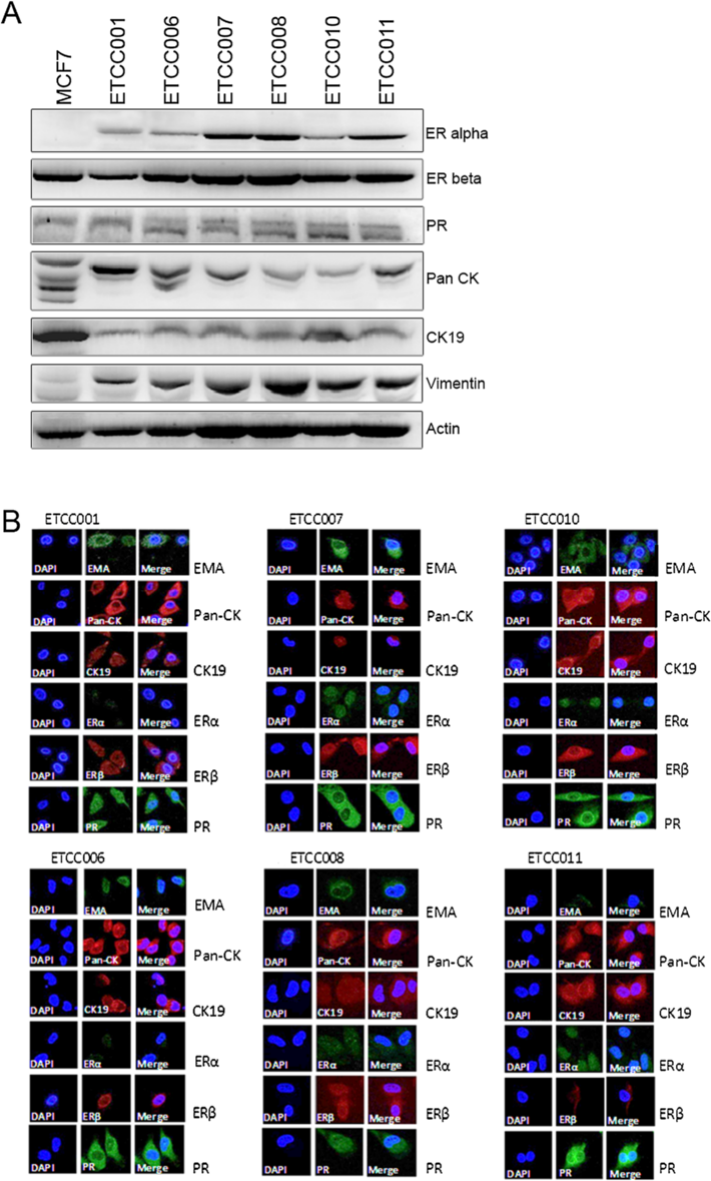
**Determination of cell markers by (A) Western blot and (B) immunofluorescence.** ETCC001 and the hTERT transfected cell lines expressed various markers. MCF7 cells were used for comparison to our DCIS cell lines. In the Western blot, the pan cytokeratins antibody could detect multiple cytokeratins in MCF7 cells but our cell lines expressed only 1–2 of these cytokeratins. DAPI was used to stain nucleus in the immunofluorescence study. Images were captured with Zeiss LSM 5 Meta microscope at 100× magnification. Pan-CK: Pan-cytokeratin; CK19: cytokeratin 19; ER: estrogen receptor; PR: progesterone receptor; EMA: epithelial membrane antigen.

We characterized the cell lines for molecular markers. Pan-cytokeratin (CK) is commonly used as an epithelial cell marker and it was used to determine whether the origin of cell lines are epithelial cells [5]. The cells were also tested for the presence of estrogen receptor (ER) and progesterone receptor (PR) as these receptors are important in determining the course of treatment of breast cancer [6]. MCF7, an epithelial breast cancer cell line, was used as the control. Western blot results (Figure 3A) showed that the six cell lines expressed pan-CK, CK-19, vimentin, ER alpha, ER beta and PR. The level of expression of these markers varies between the cell lines.

To confirm the results of the Western blots and to check for other markers for the epithelial cells, immunofluorescence assays were done (Figure 3B). The epithelial membrane antigen (EMA), an epithelial cell marker was included in addition to pan-CK [6]. EMA and CK-19 are markers for breast cancer cells [7,8]. EMA, pan-CK and CK-19 were detected in all cell lines showing that the cells are of epithelial origin (Figure 3B).

### Genomic analysis

**Figure 4:**
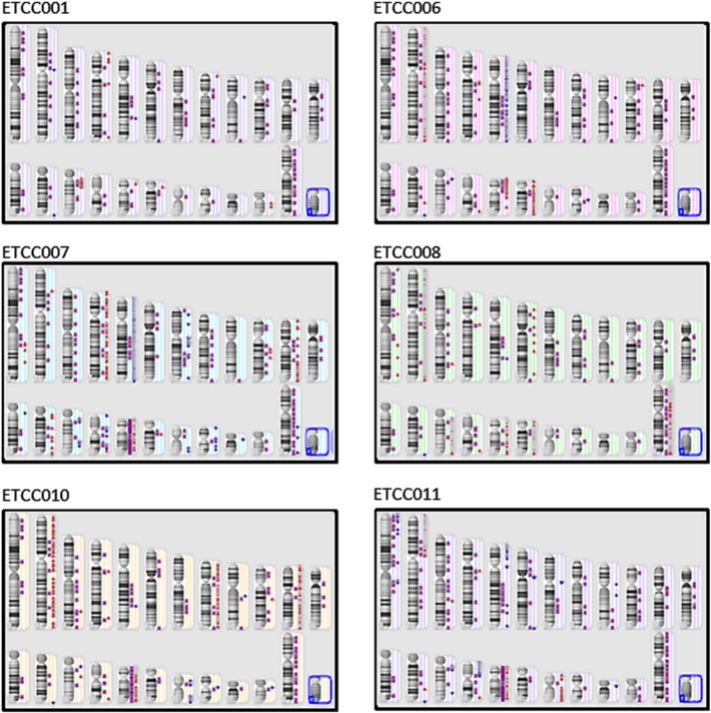
**Array CGH was used to determine chromosomal changes in the DCIS cell lines and digitally constructed karyograms are shown.** Loss of heterozygosity (LOH) is shown in purple (first column on the right of the chromosome). Red dots indicate loss and blue dots indicate gain of chromosomal materials (second and third columns on the right of the chromosome, respectively). Chromosomes are arranged from left to right (chromosomes 1 to 12 on the first row, chromosomes 13 to X on the second row). Blue boxes indicate chromosome Y which are not present in these cell lines.

Genomic analysis was performed on the cells (ETCC001 at passage 100 and the hTERT-expressing cell lines at passage 50–60) to determine if they contained genomic mutations commonly found in breast cancer. Digital karyograms documenting the gene gain, gene loss and loss of heterozygosity (LOH) are shown in Figure 4. Genetic changes of selected cancer causal gene [9,10] are shown in Table 1. LOH was generally observed in chromosome X in all the cell lines. There were significant gene gains in chromosome 5 in ETCC006 and ETCC007. This coincided with gain in *APC* (5q21) and *TERT* (5p15) gene copy numbers observed in ETCC006, ETCC007 and ETCC011. Significant LOH was observed in chromosome 17 in ETCC006, ETCC007, ETCC010 and ETCC011. Figure 3B showed a list of published cancer-causal genes identified from various primary tumor tissues [9]. CGH analysis revealed that the hTERT transformed cell lines contained more genetic changes than ETCC001. There were gene loss and/or LOH of *TP53* (17p13) and copy number neutral LOH in *MAP2K4* (17p11) in all cell lines except ETCC008. Copy number neutral LOH in *BRCA1* (17q21) is observed in ETCC007, ETCC010 and ETCC011. The gene for a regulatory subunit of protein kinase A, *PRKAR1A* (17q23), was loss or LOH in ETC007, ETCC010 and ETCC011.

**Table 1:** **Array CGH was performed to determine chromosomal changes in the DCIS cell lines**

**No.**	**Gene**	**ETCC001**	**ETCC006**	**ETCC007**	**ETCC008**	**ETCC010**	**ETCC011**	**Location**
1	*ABL1*	-	-	-	-	-	-	9q34.1
2	*ABL2*	-	-	-	-	-	-	1q24-q25
3	*ALPK2*	-	-	-	Loss(1)	-	-	18q21.31q
4	*APC*	-	Gain(3)	Gain(3)	-	-	Gain(3)	5q21-q22
5	*ATM*	-	-	-	-	-	-	11q22.3
6	*BCR*	-	-	-	-	-	-	22q11.21
7	*BMPR1A*	-	-	-	-	-	-	10q22.3
8	*BRAF*	-	-	-	-	-	-	7q34
9	*BRCA1*	-	-	CNLOH	-	CNLOH	CNLOH	17q21
10	*BRCA2*	-	-	-	-	-	-	13q12.3
11	*CDK4*	-	-	-	-	-	-	12q14
12	*CDKN2A*	-	-	-	-	-	-	9p21
13	*CHEK2*	-	-	-	-	-	-	22q12.1
14	*CREBBP*	-	-	-	-	-	Gain(3)	16p13.3
15	*CTNNB1*	-	-	-	-	-	-	3p21
16	*CTNNBIP1*	-	-	-	-	-	-	1pter-p36.31
17	*EGFR*	-	-	-	-	-	-	7p12
18	*EIF4E*	-	-	-	-	-	-	4q21-q21
19	*EP300*	-	-	-	-	-	-	22q13
20	*ERBB2*	-	-	CNLOH	-	-	CNLOH	17q21.1
21	*ETV6*	-	-	-	-	-	-	12q13
22	*FGFR1*	CNLOH	CNLOH	CNLOH	CNLOH	-	CNLOH	8p11.2-p11.1
23	*FGFR2*	-	-	-	-	-	-	10q26
24	*FGFR3*	-	-	-	-	-	-	4p16.3
25	*FHIT*	-	-	-	-	-	-	3p14.2
26	*FLT3*	-	-	-	-	-	-	13q12
27	*HRAS*	-	-	-	-	-	-	11p15.5
28	*KIT*	-	-	-	-	-	-	4q11-q12
29	*KRAS2*	-	-	-	-	-	-	12p12.1
30	*MAP2K4*	-	Loss(1)/LOH	CNLOH	-	CNLOH	CNLOH	17p11.2
31	*MAPK11*	-	-	-	-	-	-	22q13.33
32	*MAPK12*	-	-	-	-	-	-	22q13.3
33	*MAPK13*	-	-	-	-	-	-	6p21
34	*MAPK14*	-	-	-	-	-	-	6p21.3-p21.2
35	*MET*	-	-	-	-	-	-	7q31
36	*MLL*	-	-	-	-	-	-	11q23
37	*MTOR*	-	-	-	-	-	-	1p36
38	*MYC*	-	-	-	-	-	-	8q24.12-q24.13
39	*MYST3*	-	-	-	-	-	-	8p11
40	*NRAS*	-	-	-	-	-	-	1p13.2
41	*NTRK1*	-	-	-	-	-	CNLOH	14q21-q22
42	*NTRK3*	-	-	-	-	-	-	15q24-q25
43	*PAX8*	-	Loss(1)	-	Loss9(1)	-	-	2q12-q14
44	*PDGFRA*	-	-	-	-	-	-	4q12
45	*PIK3C2G*	-	-	-	-	-	-	12p12
46	*PIK3CA*	-	-	-	-	-	-	3q26.3
47	*PIP*	-	-	-	-	-	-	7q34
48	*PPARG*	-	-	-	-	-	-	3p25
49	*PRCC*	-	-	-	-	-	CNLOH	1q21.1
50	*PRKA1A*	-	-	CNLOH		CNLOH	CNLOH	17q23-q24
51	*PRKCB*	-	-	-	-	-	Gain(3)	16p12
52	*PTEN*	-	-	-	-	-	-	10q23.3
53	*RB1*	-	-	-	-	-	-	13q14.2
54	*RET*	-	-	-	-	-	-	10q11.2
55	*SMAD4*	-	-	-	-	-	-	18q21.1
56	*SCGB2A2*	-	-	-	-	-	-	11q13
57	*SCGB2A1*	-	-	-	-	-	-	11q13
58	*STK11*	-	-	-	-	-	-	19p13.3
59	*TCF4*	-	-	-	-	-	-	18q21.1
60	*TERT*	-	Gain(3)	Gain(3)	-	-	Gain(3)	5p15.33
61	*TFF1*	-	-	-	-	-	-	21q22.3
62	*TP53*	Loss(1)	Loss(1)/LOH	CNLOH	-	Loss(1)/LOH	CNLOH	17p13.1
63	*VHL*	-	-	-	-	-	-	3p25.3
64	*WWOX*	-	-	-	-	-	-	16q23.3-q24.1

Genetic changes in selected cancer causal genes are shown. Numbers in parentheses represent copy number of the gene. CNLOH: copy number neutral loss of heterozygosity.

### Mouse tumorigenicity study

**Figure 5:**
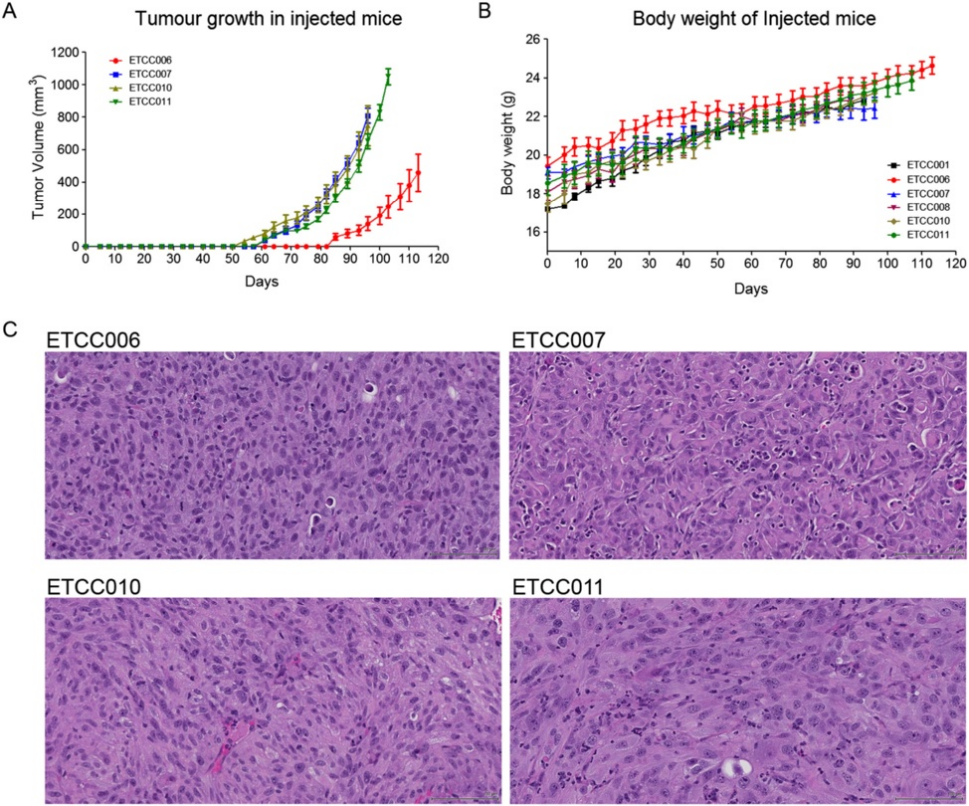
**Tumorigenicity of cell lines in SCID mice. (A)** Tumor size and **(B)** body weight of injected SCID mice were measured twice weekly. ETCC001 does not form tumor while the hTERT expressing ETCC008 form palpable tumors that were less than 30 mm^3^. **(C)** Histological images of the tumors performed at the end of the experiment show the epithelial cells arranged in densely packed solid pattern that were well differentiated with varying size and shape (H&E stained, 40X magnification).

These cell lines showed differences in their growth pattern and acceptance rate. ETCC001 and ETCC008 did not show any tumor growth and were observed to be non-tumorigenic up to 90 days. ETCC006, ETCC007, ETCC010 and ETCC011 cell lines are tumorigenic and vary in their acceptance rate and tumor growth pattern. Mice injected with ETCC011 and ETCC007 exhibited overall good acceptance (8/8) and take rate (100%) whereas the animals injected with ETCC010 and ETCC006 exhibited lower take rate of 87.5% (7/8) and 75% (6/8) respectively. Tumor volume and body weight data are represented in Figure 5A and B. Histology analyzes revealed that the epithelial cells are arranged in densely packed solid pattern and are well differentiated with varying size and shape (Figure 5C).

### Drug sensitivities of the cell lines

The cells were treated with compounds commonly used for breast cancer therapy in the clinics. In general, the cell lines were not sensitive to small molecule inhibitors paclitaxel, 5-fluorouracil, gemcitabine, cisplatin and camptothecin (Table 2). The cell lines were sensitive to doxorubicin with the exception of ETCC007 and ETCC008. The presence of the estrogen and progesterone receptors suggests that the growth of the cell lines could be regulated by hormones and the use of hormone suppressors could negatively regulate the proliferation of these cells. Indeed, our cell lines were sensitive to estrogen receptor antagonist Tamoxifen. This finding correlates well with the presence of estrogen receptor on these cell lines as demonstrated by immunostaining and Western blot results. Monoclonal antibody (Herceptin) against human epidermal growth factor receptor 2 (Her2), is not cytotoxic to the cell lines up. This may suggest that there is no Her2 amplification in the cells. Her2-negative breast cancers tend to be less aggressive [11] and this correlates well with our cell lines which are derived from a subject with breast ductal carcinoma in situ.

**Table 2:** **Drug sensitivity of ETCC001 and sub-lines**

		**IC**_**50**_ **(μM)**						
**Anti-neoplastic Class**		**ETCC001**	**ETCC006**	**ETCC007**	**ETCC008**	**ETCC010**	**ETC011**	**MCF7**
ER antagonist	Tamoxifen	6.3	34.2	32.8	32.4	30.0	33.5	26.6
Monoclonal Ab	Herceptin	>10	>10	>10	>10	>10	>10	Not Done
Anti-microtubular	Paclitaxel	>100	>100	>100	>100	>100	>100	>100
Anti-metabolite	5-fluorouracil	>100	>100	>100	>100	>100	>100	>100
Anti-matabolite	Gemcitabine	>100	>100	>100	>100	>100	>100	>100
Alkylating	Cisplatin	>100	>100	>100	>100	>100	>100	>100
Anthracycline	Docorubicin	9.3	3.2	>100	77.8	5.0	20.8	12.3
Topo I inhibitor	Camptothecin	>100	>100	>100	>100	>100	>100	4.5

ER: estrogen receptor; Ab: antibody; Topo: topoisomerase

Cells were treated with common chemotherapeutic agents and viability of the cells was determined by measuring cellular ATP levels after 48 hours. Concentration of drug (in μM) which inhibits 50% cell viability (IC_50_) is shown. The experiments were carried out in triplicates and repeated on different passages of the cells. The result shown is representative of a single experiment.

## Discussion

In this study, we have established 5 cell lines from primary cells from breast cancer tissue obtained from a Chinese female patient of Singapore origin. The primary cell line, ETCC001, is confirmed to be of human origin and free from inter-species contamination.

Cell lines are widely used in many aspect of laboratory research. It has been reported that 18% of new cell lines deposited at the German Cell Line Bank were found to be cross-contaminated [12]. It is important to ensure that the cell lines established in our laboratory are well characterized with their identities confirmed prior to using these cell lines for further research. However, tests such as isoenzyme typing, karyotyping, or DNA fingerprinting can be time consuming and costly. Using multiplex PCR, STR profiling with a standard set of STR primers has been proposed as a reliable and cost effective method for cell line identification [13]. In this report, 9 STR loci have been selected [14] and tested for their high discrimination when used in combination (1 in 10^8^ discrimination rate for unrelated individuals).

Breast tumor biopsies are heterogeneous and it is possible that the derived cell lines could be contaminated with fibroblasts and normal epithelial cells. In order to rule out these possibilities, we have performed magnetic sorting for fibroblasts and surface marker analyses of the cell lines. The cell lines exhibit typical epithelial markers (pan-CK) as well as epithelial cancer markers CK19 and EMA [7,8].

Human mammary epithelial cells have been found to stop dividing at 55–60 population doublings. Transfection of these cells with hTERT are found to extend cell division to over 250 population doublings [15]. In our case, the expression of hTERT has allowed the cell lines to grow and proliferate in culture medium RPMI1640 not supplemented with growth factors and hormones. While it appears that the level of telomerase activity in the cells does not correlate with population doubling rate, all the cells were able to reach 100% confluency in culture in contrast to ETCC001, which can only reach 50% confluency before growth stagnation. We have successfully propagated these transformed cells to high passage numbers (see Results) and are able to grow them in the RPMI1640 medium without growth factor supplements. This demonstrates that the cells are immortalized and are capable of continuous growth.

We report here the establishment of breast cell lines from non-invasive breast DCIS that express both ER and PR. Most common breast cell lines such as the MDA-MB-XXX series, BT20, SkBr3 and ZR75.1 do not express ER and PR. On the other hand, there are fewer choices of cell lines positive for the expression of ER and PR, examples being MCF-7 and T47D. Even though MCF7 and T47D express both ER and PR, they are of metastatic origin and are derived from lung pleural effusion [1]. Some recent examples of ER and PR positive breast cell lines are MBC1 and MBC2 derived from Malaysian patients [16] and BC-019, BC-020 and BC-021 from Chinese patients [17]. However, unlike our cell lines, both the Malaysian breast cell lines (MBC1 and MBC2) and the Chinese cell lines (BC-019, BC-020 and BC-021) are derived from breast tissues with invasive breast ductal carcinoma [16,17]. Other examples of breast DCIS cell lines are DCIS.COM [18] derived from ras-transformed MCF-10AT cells from fibrocystic breast tissue, SUM225 [19] derived from chest wall recurrence of a DCIS patient treated with mastectomy, and h.DCIS.01 [20] from breast columnar cell hyperplasia. Of these, only h.DCIS.01 cells are positive for ER and PR expression.

Karyotyping with GTL-banding of metaphase cells shows that ETCC001 is heteroploid with multiple chromosomal abnormalities. Our CGH analysis also shows chromosomal variations among the cell lines. We observed LOH on the X-chromosomes on ETCC001 and all its derived cell lines. LOH on the X-chromosome have been reported to be associated with human breast cancer [21,22] and other cancers [23–25]. BRCA1 participates in the maintenance of X-chromosome inactivation. The loss of the *BRCA1* wild-type allele inherited from the unaffected parent through LOH represents the event that initiates the tumorigenesis process observed in primary breast and ovarian tumors [26]. We have observed *BRCA1* copy number neutral LOH in three of the derived cell lines. Chromosome 17 is also a chromosome that suffers most LOH and/or loss of genes in all the derived cell lines. Besides *BRCA1*, other important cancer causal genes on chromosome 17 such as *TP53*, *MAP2K4*, *ERBB2* and *PRKAR1A* [9] are all observed to have loss or copy number neutral LOH in these cell lines. Deletions of functional copies of *MAP2K4* and *BRCA1* have also been reported in several breast cancer cell lines [27]. This may explain the transformation of the cells leading to immortalization. hTERT levels do not correlate to the amount of genetic aberrations observed in each cell lines. However, the amount of genetic aberrations seems to correlate with the ability of the cellsto form colonies in soft agar and tumorigenicity in SCID mice.

One of the definitive assays to determine whether cells are transformed is anchorage-independent growth. All cell lines, except ETCC001, can form colonies in soft agar. This suggests that ETCC001 is untransformed while the cell lines over-expressing hTERT are transformed. hTERT levels do not correlate with the ability of the cells to form colonies in soft agar and tumorigenicity in SCID mice. It is likely that the changes in gene copy number observed in the CGH study could have contributed to their ability to grow in the soft agar. Extended number of cell doublings through bypassing cellular senescence would allow accumulation of genetic changes favoring cell survival. Extension of lifespan by telomerase was reported to select for c-myc over-expression in human mammary epithelial cells [15]. We have shown using CGH analysis that the five cell lines generated with hTERT transformation have more genomic changes than the original cell line.

Our hTERT-transformed cell lines can form colonies in soft agar but are only weakly tumorigenic in the SCID mice. The weak tumorigenicity in SCID mice is representative of its origin as a non-invasive breast ductal carcinoma in situ. Cells derived from certain epithelial tissues have been reported to have in vitro anchorage-independent phenotype that is not associated with tumorigenicity [28]. These exceptional cells may express new cell surface antigens leading to the inhibition of tumor growth by the residual immune response or non-specific killer lymphocytes [29] in the SCID mice. These observations led to the hypothesis that the loss of the anchorage requirement in vitro, for example in the soft agar assay, is a necessary but not a sufficient correlation of cellular malignancy.

Sensitivities toward tamoxifen and doxorubicin have decreased in the hTERT-expressing cell lines compared to the primary ETCC001 cell line. This could be due to more genetic aberrations in the hTERT-expressing cell lines leading to loss of tumor suppressors and/or activation of oncogenes as discussed above. However, this does not imply a loss of the DCIS identity as we still observed weak tumorigenicity in SCID mice with these hTERT-expressing cell lines which is consistent with the non-invasive nature of DCIS in early phase breast cancer.

## Conclusions

We have established five cell lines from a patient with breast ductal carcinoma in situ. The cell lines were derived from cells that have been stably transformed with hTERT. A compendium of genetic, physical and molecular characterizations has demonstrated that the five cell lines carry distinct genetic changes. The cell lines also express estrogen and progesterone receptors, CK19, EMA and other surface biomarkers, confirming that they are of breast epithelial origin. The cell lines together with the information that we have acquired will provide a rich resource in drug discovery and cell physiology studies for early stage breast cancer.

## Methods

### Cancer tissues and reagents

Tissue was obtained from breast ductal carcinoma in situ (DCIS) of a 47 year-old female of Chinese origin. The Cancer Cell Isolation Kit (Panomics) was used to obtain primary cells from the tissue. Fibroblasts were removed using the MAC Separator (Miltenyi Biotech). Primary cells obtained were labeled as ETCC001 and were grown in M171 media (Life Technologies) with mammary epithelial growth supplement (MEGS). Cell lines arising from ETCC001 after hTERT transformation were grown in RPMI supplemented with 10% fetal bovine serum (FBS), L-glutamine and Penicillin/Streptomycin (Life Technologies). Cell cultures were maintained in a humidified incubator at 37°C with 5% atmospheric CO_2_.

### hTERT transfection and generation of stable clones

pGRN145 vector containing hTERT sequence (ATCC) and hygromycin resistant gene was used to transfect ETCC001 primary cells. Transfection was performed using Lipofectamine 2000 (Life Technologies). 30 μg/ml of Hygromycin (Life Technologies) was used to select for positively transfected cells. 1 × 10^3^ hygromycin-resistant cells were seeded in a 15-cm petri dish and grew for at least 2 weeks. Single cell colonies were then amplified. Telomerase activity in these cell colonies was measured using the TRAPeze Telomerase Detection Kit (Millipore). Heat inactivated samples were used as negative controls for the telomerase activity detection.

### Cell line authentication and virology safety testing

ETCC001 cells in metaphase were fixed and stained for Giemsa/Trypsin/Leishman (GTL)-banding to obtain karyograms (Cytogenetics laboratory, National University Hospital, Singapore). Cell pellets were also sent to IDEXX Laboratories Inc (Columbia, Minnesota) for further authentication and virological safety testing. Short tandem repeat (STR) analysis were done to establish cell line identity, inter-species (rat, mouse, Chinese hamster, African Green Monkey). The cell lines were tested for contaminations by polymerase chain reaction (PCR) screening for 18 types of virus, and mycoplasma.

### Western blot and immunofluorescence staining for cell markers

Western blot and Immunofluorescence staining were performed to detect breast and epithelial cell markers. Primary antibodies used were mouse anti-EMA (Dako), rabbit anti-Pan-Ck (Abcam), rabbit anti-Ck19 (Abcam), mouse anti-estrogen receptor (ER) α (Abcam), rabbit anti-ERβ (Abcam) and mouse anti-progesterone receptor (PR) (Abcam). Secondary antibodies used in Western blot analysis were horse-radish peroxidase conjugated goat anti-rabbit and anti-mouse antibodies (Dako). Secondary antibodies used in immunofluorescence study were Alexa Fluor 594 goat anti-rabbit (Life Technologies) and Alexa Fluor 488 goat anti-mouse (Life Technologies). Antibody concentrations used in Western blot and immunofluorescence staining were as recommended by the antibody manufacturers.

### Cell doubling time

Cell doubling time was determined using the IncuCyte real-time cell analyzer (Essen Bioscience). The Incucyte uses cell confluency as a measure of cell growth. 1 × 10^5^ cells were seeded in T-25 flasks and placed in the IncuCyte. Cell growth was monitored until confluency was achieved. Analysis was performed with the IncuCyte software which auto-generates growth curve according to cell confluency.

### Comparative genomic hybridization (CGH) and karyotyping

Cell pellets containing 1 × 10^6^ cells were sent to Origen Labs (Singapore) for CGH array hybridization using the Affymetrix SNP 6.0 platform. Data analysis was performed with Affymetrix Chromosome Analysis Suite.

### Soft agar assay

Soft agar colony formation assay was done in 24-well plates. Each well contained 0.6 ml of 0.6% agar (Sigma) in complete medium in the bottom layer, 0.5 ml of 0.36% agar in complete medium with cells in the middle layer, and covered with 0.5 ml medium. The cells were cultured at 37°C with 5% atmospheric CO_2_ for 2 to 3 weeks. After overnight staining with tetrazolium dye, MTT (Sigma) at 70 μl per well at 5 mg/ml, the colonies were counted using the GelCount® instrument (Oxford Optronix).

### Mouse tumorigenicity study

10 × 10^6^ cells from ETCC001 and the five hTERT-expressing cell lines were injected subcutaneously into the right flank of each female SCID mice (age 6–8 weeks). Eight mice were injected for each cell lines. All the animals were observed for clinical signs, body weight, tumor volume & mortality. Clinical signs, body weight and tumor volume were recorded twice weekly throughout the experiment. The mice were sacrificed at the end of the experiment. A piece of each palpable tumor was snap frozen or fixed in 10% Neutral-buffered formalin solution for histopathology study by hematoxylin and eosin (H&E) staining. The study was carried out at the A-STAR Biological Resource Center - an Association for Assessment and Accreditation of Laboratory Animal Care (AAALAC) accredited facility. All animals were handled humanely with due regard for their welfare. The study was designed to use the fewest possible number of animals. The study design was reviewed and approved by the Institutional Animal Ethics Committee.

### Drug treatment

Tamoxifen, paclitaxel, 5-fluorouracil, gemcitabine, cisplatin, doxorubicin and camptothecin were purchased from Calbiochem, and herceptin was purchased from Roche. Cells were seeded in 50 μl medium in 96-well plates at 8000 cells/well and incubated overnight. Compounds (50 μl) were added to cells and incubated for 48 hours.100 μl of CellTiter-Glo (Promega) was added to the cells. After 10 minutes of incubation at room temperature, luminescence was measured with Safire II plate reader (Tecan). Data was analyzed with Graphpad Prism software and the half maximal inhibitory concentration (IC_50_) was determined. Error bars denote standard deviation (SD).

## Abbreviations

AAALAC: Association for Assessment and Accreditation of Laboratory Animal Care
A-STAR: Agency for Science Technology and Research
CK19: Cytokeratin 19
DCIS: Ductal carcinoma in situ
EMA: Epithelial membrane antigen
ER: Estrogen receptor
GTL: Giemsa/trypsin/Leishman
H&E: Hematoxylin and eosin
Her2: Epidermal growth factor receptor 2
hTERT: Human telomerase reverse transcriptase
CGH: Comparative genomic hybridization
LOH: Loss of heterozygosity
Pan-CK: Pan-cytokeratin
PCR: Polymerase chain reaction
PR: Progesterone receptor
SD: Standard deviation
STR: Short tandem repeat
